# Diallel analysis of soybean (*Glycine max* L.) for biomass yield and root characteristics under low phosphorus soil conditions in Western Ethiopia

**DOI:** 10.1371/journal.pone.0281075

**Published:** 2023-02-06

**Authors:** Abush Tesfaye Abebe, Samuel Adewale, Godfree Chigeza, John Derera

**Affiliations:** 1 International Institute of Tropical Agriculture, Ibadan, Nigeria; 2 International Institute of Tropical Agriculture, Lusaka, Zambia; KGUT: Graduate University of Advanced Technology, ISLAMIC REPUBLIC OF IRAN

## Abstract

Combining ability studies under low soil P conditions provides useful information on the inheritance of important traits to improve soybean for low P tolerance. The study aimed at determining the combining ability and gene actions of biomass yield and root traits in soybean under low phosphorus conditions. Nine parental genotypes and their 36 half diallel F2/F3 progenies were evaluated at two locations in Ethiopia on soils of low P availability. Highly significant (P<0.01) general combining ability (GCA) were found for all the traits and specific combining ability (SCA) for root dry weight and root fresh weight; while the SCA effects of all the rest of the traits were significant (P<0.05). The higher relative contributions of GCA over SCA revealed the preponderance of additive gene action in the inheritance of biomass yield, root dry weight, biomass dry weight, root volume, and root fresh weight with respective relative GCA:SCA contributions of (60.6, 39.4), (50.4, 49.6), (54.9, 45.1), (51.1, 48.9), and (52.1, 47.9); while the narrow-sense heritability was high (34.3%) only for biomass yield. Hardee-1 displayed significant (P<0.05) and positive GCA effects for most of the studied traits, and several crosses involving this parent showed superior performances. The traits i.e., biomass yield, biomass dry weight, root volume and root fresh weight showed highly (P<0.001) correlation with grain yield. Thus, breeding programs aiming to improve soybean for biomass yield and root traits under low-P condition can use Hardee-1 as a parent.

## Introduction

Soybean (*Glycine max* L. Merril) is one of the world’s most economically important legumes and oilseeds, and its global production and yield have increased considerably in the last three decades [1]. It is an essential source of protein, vegetable oil, and micronutrients produced both commercially and under subsistence farming conditions, primarily used for human food and animal feed. The soybean biomass has great potential as a high protein animal feed in the form of graze, hay or silage [2–4].

Soybean provides a pivotal ecological function in the cropping system, including the diversification of crops in the agro-ecosystems, decreasing soil-borne diseases, breaking the pest and disease cycles in cropping systems of continuous cereals production, improving soil nitrogen fixation, soil P availability and soil carbon sequestration [5–10].

Despite the rapidly growing trend of annual production and demand of the crop, both the grain and biomass yield of soybean were low in most soybean growing agro-ecologies of Ethiopia. Low phosphorus availability in the soil, caused due to soil acidity, which covers an estimated more than 50% of the global [11] and 28% of the Ethiopian [12, 13] arable land is among the major soybean production constraints affecting the biomass/forge yield of soybean in tropical soils [14]. Phosphorus forms insoluble compounds in acid soils reacting with other nutrients, such as aluminum and iron, converting it into a form that is not usable by plants [15].

Phosphorus (P) is among the essential soil nutrients required in large quantities for crop growth and development, throughout their life cycle, especially during the reproductive and seed development stages. However, most tropical and sub-tropical soils that lack proper fertilization and manuring treatments do not contain sufficient readily available P to meet the high demands of the crop, especially during certain periods of the growing cycle [16]. Thus, crop production in these regions generally depends on the timely application of P fertilizer. Development of P-efficient soybean varieties that can efficiently utilize the native P and added P in the soil would be a feasible and sustainable approach to boost soybean production and forage yield [14].

In soybean, low P stress is more detrimental than other nutrient deficiencies, toxicities or diseases; however, it is difficult to select high P efficient soybean varieties based on phenotypes. The soybean demand for P reaches the maximum during pod and seed development [17, 18], as more than 60% of the P ends up in the pods and seeds [17]. About 39% of P uptake takes place during the R4 (full pod stage) in soybean that is the critical stage for seed yield [18]. Its uptake and utilization by soybean is essential in ensuring proper nodule formation, as well as crop yield and quality improvement [19], as it plays a very important role in the storage and transportation of energy generated in photosynthesis [18]. Remarkably, high soil phosphate depressed seed protein and oil content, while yield would be low, if available phosphorus was less than 30 kg P ha^-1^ [20]. Phosphorus is the most important nutrient in forage biomass yield, followed by nitrogen, as high biomass production extracts high amount of P from the soil and P improves biomass production in forages [14]. Suman [18] reported association of biomass yield with nutrient accumulation in soybean.

Soybean is primarily produced for grain purpose, however, in the 1800’s or before world war-II it used to be cultivated mainly for forage purposes in the United States [2, 4]. Moreover, soybean has great future potential as a high protein and nutritious forage in the form of pasture, hay or silage that can be used alternatively with alfalfa, possessing equivalent forage quality [2, 4]. Unlike other legume crops, soybean leaves and pods provide highly digestible protein and energy, when the soybean is closer to maturity, before yellowing of the leaves and at 90% pods fill [2, 4]. Especially, in seasons of terminal moisture stress the soybean crop that failed to attain proper grain filling due to moisture stress can be used as a very good forage [2]. Moreover, studies verified the possibility of harvesting more than 9.88 t ha^-1^ of forage from soybean [3]. Varietal difference was identified as one of the factors affecting forage quality, and forage soybean varieties were exclusively developed and registered in Minnesota [21]. One IITA soybean variety, TG X1990- 114FN was also registered as a forage variety in Ethiopia, after verified for its nearly the same nutritional quality and high invitro digestibility with Lablab.

Plant root traits play important roles in increasing soil P availability and acquisition and adaptation to soil P deficiency. Root characteristics such as high root biomass, length and volume enable the plants to occupy greater soil volume and enhance P acquisition by plants [10]. Root distribution and architecture have critical roles in optimizing the absorption of soil resources in specific environments, for instance under low P stress conditions [22, 23]. Greater number of basal root whorls and hypocotyl-borne roots reported increasing the total root length in the topsoil resulting in greater P acquisition in common bean [24].

Combining ability studies provide valuable information that helps in making decision on the choice of parental line and allows understanding the gene action influencing trait inheritance resulting in greater efficiency in soybean breeding programs [1]. Plants with tolerance to low*‐*P can grow better under low*‐*P conditions, and understanding the genetic mechanisms of low*‐*P tolerance will not only facilitate identifying the relevant genes, but also helps in developing low*‐*P tolerant cultivars [16]. Breeding crop cultivars that could uptake/utilize soil P more efficiently is critical in improving the productivity of soybean under low P conditions and increasing resource use efficiency in agriculture. Unpredictable growth environments, decreasing moisture availability, altered precipitation patterns, ongoing soil degradation, and the rising cost of nitrogen and P fertilizers are some of the key reasons for developing crop varieties resilient to abiotic stresses [23, 25]. Even though root traits associated with shoot traits contributing to productivity have been identified in soybean [26], their beneficial role in breeding for yield improvement is yet to be exploited. Therefore, the objectives of this study were to determine the gene actions controlling fresh biomass yield, dry matter accumulation, and root characteristics under low P conditions in soybean.

## Materials and methods

### Germplasm

The parental lines (Table 1) used in this study included four varieties (Clark 63 K, Crowford, Davis, and SCS-1) released for high yield and adaptability in the mid-altitude (1400–1900 masl) soybean growing agro-ecologies of Ethiopia, and five of the parents i.e., Hardee-1, Alamo, PR-142 (26), H 3, and G 9945 were identified as low P tolerant genotypes in a screening experiment involving 36 soybean genotypes evaluated under low (zero) and high (100 kg ha^-1^) applied P conditions. The parental line, H 3, was received from Mozambique Agricultural Research Institute, was specifically identified for its low P tolerance. All the parental lines used in this study had been tested in yield trials for over three years and in at least six locations in each year and showed good performance and adaptability for the mid-altitude and high rainfall soybean growing agro-ecologies of the country. While being among the parental lines, the released varieties (Clark 63 K, Crowford, Davis and SCS-1) might serve as standard checks for the tested genotypes in this experiment. The nine parental lines were crossed in a 9 x 9 half diallel mating scheme. The diallel experiment was conducted using F2/F3 progenies due to inadequate quantities of seeds in the F1.

**Table 1:** Characteristics of the nine soybean parental genotypes used in the present study.

Genotype	Low P tolerance	Farmers’ preferred agronomic features
Hardee -1	Tolerant	High yielding, well adapted to mid-altitude environments
Davis	Not known	High yielding, well adapted to mid-altitude environments
Alamo	Tolerant	High yielding, well adapted to mid-altitude environments
PR-142 (26)	Tolerant	High grain yield, high biomass
H 3	Tolerant	High yielding, well adapted to mid-altitude environments
Clark 63 K	Not known	Released variety, high yielder, and well adapted
SCS-1	Not known	Released variety, high yielder, and well adapted
G-9945	Tolerant	High yielding, well adapted to mid-altitude environments
Crowford	Not known	Released variety, high yielder, and well adapted

### Experimental design and management

The diallel trials consisting of 45 genotypes (parents and progenies) were grown at two locations, i.e., Assossa (altitude 1550 m.a.s.l, location 10°02’N34°33’E), and Metu (altitude 1550 m.a.s.l., location 8°3’ N 30°E) in Western Ethiopia in a 5 x 9 alpha lattice design with two replications. The two experimental sites were known for their strongly acidic reddish-brown soil with low P availability (Table 1). Each plot consisted of four rows, of which the middle two rows were harvestable each with 4 m length, and 60 cm X 5 cm inter and intra-row spacings. Uniform application of all the best soybean management practices such as good land preparation, planting at the right sowing time, thinning to maintain a spacing of 5 cm between plants at five leaves stage, optimum weed management during the growing period were followed. Rhizobium inoculum was applied to the seeds of all the experimental materials (parents and progenies).

**Fig 1:**
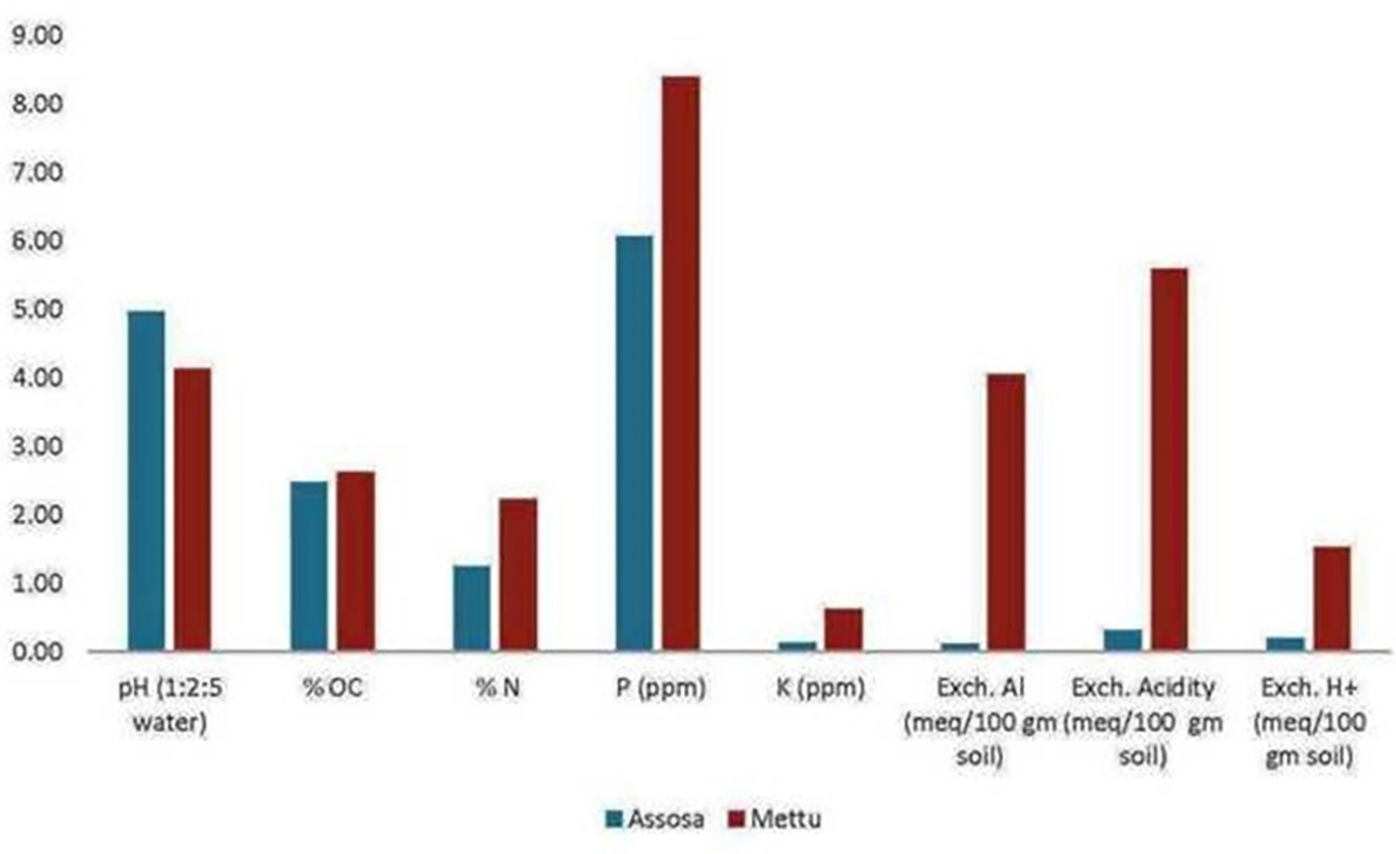
The different characteristics of the soil types of each of the test locations where the experiments were conducted.

Before the experiment, three soil samples were collected from the top layer (0–20 cm) of the fields in each of the study locations. The soil samples were submitted to the soil laboratory of Jimma Agricultural Research Center for the different analyses. Bray II method was used for P analysis, while Kjeldhal method for N, flame photometry for K and Walkley and Black method was used to determine organic carbon (OC) and organic matter (OM). Other soil parameters such as pH, exchangeable acidity, Al and H were also determined for the soil samples. Results of the soil analysis at the experimental sites (Assosa and Mettu), where this research was conducted are presented in Fig 1. Phosphorus levels of 6.07 ppm and 8.40 ppm were recorded at Assosa and Mettu, respectively.

Data were collected for biomass yield and other root traits at R6 growth stage of the crop, including biomass yield (gm): the weight of fresh above ground shoot biomass samples collected from five representative plants, while biomass dry weight (gm), which is the weight of oven dried above ground shoot biomass collected from five representative sample plants. Other root parameters collected from five randomly selected representative plants included: root fresh weight (gm) the fresh weight of root samples, root dry weight (gm) which is the weight of oven dried root samples, root length (cm) the mean length of the central longest tap roots, and root volume (ml) the volume of water displaced from a measuring cylinder by the roots.

### Statistical analysis

Analysis of variance for the individual locations was performed for each of the traits, and highly significant differences were found among genotypes for all the studied traits. Test of homogeneity of error variances of the individual locations (Assossa and Mettu) was performed for all the traits as suggested in [27] and there was non-significant differences among the error mean squares, indicating the possibility of proceeding with the combined analysis. Combined analysis of variances was performed for biomass and root traits using SAS 9.3 statistical software [28]. GCA and SCA effects were determined across the test locations using Griffing’s method II, model I, based on the Diallel SAS 05 program [29]. LSD at 5% level of significance was used for mean separation. The model for the diallel analysis for Griffing’s Method II, Model I (modified for over locations analysis from [30] is:

$$X_{ij}=X+g_{i}+gj+S_{ij}+L_{k}+{\left(gL\right)}_{ik}+{\left(gL\right)}_{jk}+{\left(SL\right)}_{ijk}+e_{ijk}/b$$

Where, *X* = the population mean, gi = the general combining ability effect of the i^th^ parent, gj = the general combining ability effect of the j^th^ parent, Sij = the specific combining ability effect of the cross between i^th^ and j^th^ parents, such that Sij = Sji, (gL)ijk = the interaction effect, if general combining ability of i^th^ parent with K^th^ locations, (gL)jk = the interaction effect of general combining ability of j^th^ parent with K^th^ locations, (SL)ijk = the interaction effect of the specific combining ability with locations, and eijk is the residual associated with ijk^th^ observation. The formula provided in [31, 32] were used to compute the variance components of GCA and SCA, the additive and non-additive variances, and heritability. The relative importance of GCA and SCA was determined using the equation:

$$\frac{2\sigma^{2}gca}{2\sigma^{2}gca+\sigma^{2}SCA}$$

*2σ*^*2*^ *gca* is the variance of GCA and σ^*2*^*sca* is the variance of SCA. Since the total genetic variance among F1 hybrids is equal to twice the GCA component plus the SCA component, the closer the ratio is to unity, the greater the predictability of a specific hybrid’s performance based on GCA alone [33, 34]. Pearson’s correlation analysis among the studied traits was performed using performance analytics package in R.

## Results

The analysis of variance revealed highly significant (P<0.01) differences among the genotypes (G), environments (E) for all the studied biomass yield and root related traits (Table 2). Similarly, GCA showed highly significant (P<0.01) differences for all the studied traits, while SCA showed highly significant (P<0.01) for only root dry and fresh weights. The SCA effects of the other traits, i.e., shoot dry weight, root length, root volume and biomass fresh weight were only significant at 5% level of significance. The G × E mean square was significant (P<0.05) for only root length, while all the other traits were non-significant. The GCA × E mean squares was highly significant (P<0.01) only for shoot dry weight and significant (P<0.05) only for plant fresh weight. The SCA× E mean squares was significant (P<0.05) only for root length.

**Table 2:** Mean squares for root traits of 45 soybean genotypes (parents and progeny populations) evaluated under low P conditions.

Source of variation	Df	Root dry weight (gm)	Shoot dry weight (gm)	Root length (cm)	Root vol (ml)	Root fresh wt (gm)	Biomass yield (kg ha^-1)^
Environment (E)	1	100.95**	38421.38**	33.06*	158.67**	331.57**	174626.90**
Rep (E)	2	40.08**	12417.58**	133.33**	272.36**	291.32**	78573.44**
Genotype (G)	44	3.19**	906.91**	14.28**	41.87**	37.21**	7113.42**
G × E	2	1.36	491.76	11.69*	18.91	10.95	3417.90
GCA	8	5.91**	1889.23**	22.35**	78.93**	72.03**	17018.50**
SCA	36	2.59**	688.62*	12.48*	33.63*	29.48**	4912.3*
GCA × E	8	1.71	989.92**	7.85	12.40	12.51	6171.3*
SCA × E	36	1.29	381.06	12.54*	20.36	10.60	2806.02
Error		1.26	403.23	7.41	20.49	12.05	2923.92
*σ* ^*2*^ *gca*		0.03	20.00	0.16	0.25	0.25	124.67
*σ* ^*2*^ *sca*		0.27	79.68	3.99	6.48	2.22	586.71

The mean performance of 66 crosses and nine parental lines evaluated under low P conditions are presented in Table 3. In addition, biomass yield differed among the parental genotypes with PR 142 (26) having the lowest value of 71.9 gm and Crowford the highest value of 168.8 gm, and a mean of 125.5 gm. The parental lines SCS-1, Hardee 1, and G-9945 produced 153.0, 148.7 and 146.8 gms of biomass, respectively. The lowest value of 42.6 gm biomass was produced by a cross of Davis x Alamo; while the highest value of 206.9 gm was recorded for Hardee-1 x Pr-142 (26). The other crosses with high biomass yield were Hardee-1 X Clark 63 (205.6 gm), Hardee-1 X Crowford (195.6 gm), and Hardee-1 X G-9945 (192.8 gm). Root dry weight among the parental genotypes varied from 2.0 gm for PR 142 (26) to 4.8 gm for Crowford with a mean of 3.3. Other parental lines with high root dry weight included: G 9945 (4.1 gm), SCS-1 (3.8 gm), Clark 63 K (3.5 gm) and Davis (3.4 gm). Similarly, the root dry weight among the crosses varied from 1.2 gm for Davis x Alamo to 4.5 gm for Hardee-1 x Clark 63 K, with an average of 2.9 gm. Hardee-1 X Pr-142 (26) (4.3 gm), Hardee-1 X SCS-1 (4.3 gm), and Clark 63 K X Crowford (4.2 gm) were some of the crosses with high root dry weight. The biomass dry weight varied from 28.2 gm for H 3 to 66.9 gm for Crowford, among the parental genotypes, with a mean of 47.5 gm. Some of the other parental lines with high biomass dry weight includes: SCS-1, G 9945, and Hardee-1 with respective weights of 55.6, 52.8 and 52.6gms. The biomass dry weight yield for the crosses varied from 18.3 gm for Pr-142 (26) x Crowford to 76.5 gm for Hardee-1 x Clark 63 K, with a mean of 40.4 gm. Furthermore, root length among the parental genotypes ranged from 14.2 cm for PR 142 (26) to 19.0 cm for SCS-1, with a mean of 17.4 cm. H 3, Crowford and G 9945 were among the parental lines that showed long root lengths of 18.4, 18.2 and 18.0 cms, respectively. The root length among the crosses ranged from 12.6 cm for Davis x Alamo to 21.4 cm for H-3 x SCS-1, with an average of 16.9 cm. The crosses with the longest root length include: Alamo X H-3 and Pr-142 (26) X SCS-1 (19.2 cm each), Clark 63 K X Crowford and Alamo X Clark 63 K (19.1 cm each). Root volume among the parental genotypes varied from 7.8 ml for H 3 to 16.5 ml for each of Crowford and G-9945 with a mean of 13.0 ml. The other parental lines with high root volume include: SCS-1 (15.5 ml), and Hardee-1 (14.0 ml). The root volumes of the crosses ranged from 6.8 ml for Davis x Alamo to 17.5 ml for Hardee-1 x G-9945 with a mean of 11.3 ml. Hardee-1 X Crowford (16.5 ml), Hardee-1 X Pr-142 (26) (16.0 ml) and Clark 63 K X SCS-1 (16.0 ml) were among the crosses that produced high root volume. Root fresh weight among the parental genotypes ranged from 6.5 gm for PR 142 (26) to 16.1 gm for Crowford, with a mean of 10.7 gm. G-9945 and SCS-1 were two of the other parental lines that produced relatively high root fresh weights of 13.4 gm and 12.9 gm, respectively. The lowest root fresh weight of 4.0 gm and the highest root fresh weight of 14.8 gm were obtained for Davis x Alamo and Hardee-1 x Clark 63 K, respectively, with 8.9 gm mean root fresh weight of the crosses. Other crosses such as Clark 63 K X Crowford, Clark 63 K X SCS-1 and H-3 X SCS-1 produced respective root fresh weights of 14, 13.1 and 13.0 gm.

**Table 3:** Mean values of root agronomic traits of soybean parental genotypes and their crosses under low P conditions.

Genotype	Root dry weight (gm)	Shoot dry weight (gm)	Root length (cm)	Root vol (ml)	Root fresh wt (gm)	Biomass yield (kg ha^-1^)
Parents						
Crowford	4.8	66.9	18.2	16.5	16.1	168.8
SCS-1	3.8	55.6	19.0	15.5	12.9	153.0
Hardee 1	3.2	52.6	17.8	14.0	10.5	148.7
G-9945	4.1	52.8	18.0	16.5	13.4	146.8
Clark 63 K	3.5	50.3	17.7	12.5	10.3	139.1
Davis	3.4	50.5	17.7	13.8	10.6	130.6
Alamo	2.6	38.2	15.7	12.3	8.2	94.3
H 3	2.5	28.2	18.4	7.8	7.4	76.5
PR 142 (26)	2.0	32.5	14.2	8.0	6.5	71.9
Parents mean	3.3	47.5	17.4	13.0	10.7	125.5
Crosses						
Hardee-1 X Pr-142 (26)	4.3	75.5	18.8	16.0	12.9	206.9
Hardee-1 X Clark 63 K	4.5	76.5	16.9	17.3	14.8	205.6
Hardee-1 X Crowford	4.0	71.6	19.0	16.5	12.7	195.6
Hardee-1 X G-9945	4.0	70.4	17.3	17.5	12.3	192.8
Hardee-1 X Alamo	2.8	62.5	15.4	12.0	8.6	155.6
Davis X SCS-1	2.7	59.2	17.9	11.3	7.9	147.6
H-3 X SCS-1	4.0	44.6	21.4	14.3	13.0	138.2
Hardee-1 X SCS-1	4.3	48.5	17.3	14.0	12.7	125.7
Davis X H-3	3.7	47.7	18.8	12.8	11.3	125.6
Hardee-1 X H-3	2.8	41.6	15.0	9.5	7.8	120.5
SCS-1 X G-9945	3.0	42.4	17.7	13.3	11.5	119.8
Clark 63 K X SCS-1	4.0	39.7	16.9	16.0	13.1	112.9
H-3 X Crowford	3.4	38.8	18.8	13.5	10.6	111.5
Clark 63 K X Crowford	4.2	39.4	19.1	14.0	14.0	108.6
Davis X Crowford	2.4	49.4	14.9	9.5	6.3	106.9
H-3 X G-9945	3.0	36.2	16.6	10.5	9.3	106.6
Alamo X H-3	3.7	37.8	19.2	14.3	11.6	103.9
Hardee-1 X Davis	2.5	35.9	17.9	11.5	7.9	101.5
Pr-142 (26) X H-3	2.7	37.5	15.8	11.8	8.3	101.0
H-3 X Clark 63 K	3.2	33.2	18.8	13.5	10.6	98.4
Pr-142 (26) X G-9945	2.6	34.0	15.0	11.0	8.9	95.7
SCS-1 X Crowford	2.6	42.5	16.0	11.0	8.6	93.7
Pr-142 (26) X Clark 63 K	2.6	32.4	16.1	8.5	7.5	91.6
Davis X G-9945	2.5	34.6	17.6	10.0	7.9	90.5
Alamo X G-9945	2.3	30.5	16.9	10.5	7.0	81.3
Davis X Pr-142 (26)	2.7	36.4	15.3	7.5	6.0	78.1
Pr-142 (26) X SCS-1	2.5	29.2	19.2	7.3	5.6	76.0
Alamo X Clark 63 K	2.2	27.5	19.1	8.3	6.9	75.0
Alamo X Pr-142 (26)	2.0	33.1	14.9	8.0	6.7	71.5
Alamo X SCS-1	1.8	39.5	13.1	7.5	5.6	67.4
Clark 63 K X G-9945	1.7	23.3	18.0	7.0	5.3	63.8
G-9945 X Crowford	2.0	19.4	15.2	9.0	6.9	60.1
Davis X Clark 63 K	1.6	27.0	13.9	10.5	5.7	58.1
Pr-142 (26) X Crowford	2.0	18.3	17.3	7.3	6.4	54.5
Alamo X Crowford	1.6	21.6	14.5	7.3	4.5	44.6
Davis X Alamo	1.2	18.4	12.6	6.8	4.0	42.6
Crosses mean	2.9	40.4	16.9	11.3	8.9	106.4

Estimates of GCA effects of root traits for the nine soybean parental genotypes evaluated under low P conditions are presented in Table 4. Hardee-1 was the only parent with highly significant (P<0.001) and positive GCA effect for dry biomass weight, and fresh biomass weight, and significant (<0.05) and positive effect for root fresh weight. The GCA estimates for Alamo showed significant negative GCA effects for all the traits, except shoot dry weight. PR 142 (26) displayed significant negative GCA effects for root volume and root fresh weight.

**Table 4:** General combining ability effects of root traits of nine soybean parental genotypes evaluated under low P conditions in Ethiopia.

	Root dry weight (gm)	Shoot dry weight (gm)	Root length (cm)	Root volume (lt)	Root fresh weight (gm)	Biomass yield (kg)
Hardee 1	0.65	24.81**	0.06	2.54*	1.91	70.42**
Davis	-0.49	-1.14	0.63	-0.91	-1.82	-5.21
Alamo	-0.85*	-9.60	-1.73*	-2.55*	-2.73**	-36.94*
PR 142 (26)	-0.49	-8.73	1.62	-2.41*	-2.05*	-23.67
H 3	0.37	-7.19	22.43	-0.41	0.64	-9.98
Clark 63 K	0.19	-3.46	0.02	0.49	0.68	-1.58
SCS-1	0.37	8.99	11.64	1.45	1.68	14.79
G-9945	-0.26	-6.78	1.16	0.59	0.05	-13.30
Crowford	0.21	1.47	0.12	0.44	0.88	0.99
S.E	0.23	13.65	0.46	2.27	0.61	10.54

Among the crosses, Hardee-1 x H-3, Davis x Clark 63 K, Alamo x SCS-1 showed significant negative SCA effects for root length, whereas Alamo x Clark 63 K showed significant positive SCA effects for root length (Table 5). Similarly, Hardee-1 x H-3 and Clark 63 K x G-9945 displayed significant negative SCA effects for root volume. In addition, Hardee-1 x Pr-142 (26) showed significant positive SCA effect for plant fresh weight.

**Table 5:** Specific combining ability effects of root traits of 45 soybean progeny populations evaluated under low P conditions in Ethiopia.

Genotype	Root dry Weight (gm)	Shoot dry Weight (gm)	Root length (cm)	Root Volume (lt)	Root fresh Weight (gm)	Biomass Yield (kg)
Hardee-1 x Davis	-0.66	-20.50	1.12	-1.68	-1.71	-45.92
Hardee-1 x Alamo	-0.10	11.79	-0.74	-0.43	-0.35	24.63
Hardee-1 x Pr-142 (26)	1.15	23.47	2.38	4.10	3.55	67.88*
Hardee-1 x H-3	-0.86	-11.53	-3.34*	-4.43*	-3.51	-31.20
Hardee-1 x Clark 63 K	0.84	20.96	-0.75	2.91	3.33	50.90
Hardee-1 x SCS-1	0.58	-12.24	-0.65	-0.84	0.74	-37.67
Hardee-1 x G-9945	0.52	15.17	-0.02	3.00	1.07	37.02
Hardee-1 x Crowford	0.25	12.9	1.56	2.07	0.95	38.95
Davis x Alamo	-0.79	-16.14	-2.72	-2.50	-2.04	-34.76
Davis x Pr-142 (26)	0.46	0.54	-0.36	-1.22	-0.36	-7.28
Davis x H-3	0.88	10.71	1.28	2.00	2.90	27.49
Davis x Clark 63 K	-1.17	-12.32	-2.95*	-0.65	-2.81	-43.01
Davis x SCS-1	-0.19	14.66	0.75	-0.40	-1.04	37.82
Davis x G-9945	-0.12	-4.38	1.12	-1.31	-0.37	-11.74
Davis x Crowford	-0.47	6.9	-1.69	-1.75	-2.51	3.86
Alamo x Pr-142 (26)	0.07	2.93	-0.10	0.03	0.92	2.65
Alamo x H-3	1.16	6.45	2.38	4.25	3.81*	22.37
Alamo x Clark 63 K	-0.24	-6.12	2.90*	-2.15	-0.98	-9.61
Alamo x SCS-1	-0.83	0.68	-3.39*	-3.40	-2.74	-25.90
Alamo x G-9945	-0.04	-2.89	1.00	-0.06	-0.64	-4.42
Alamo x Crowford	-0.96	-15.3	-1.46	-3.25	-3.68	-41.94
Pr-142 (26) x H-3	-0.04	4.90	-1.35	2.28	0.19	11.42
Pr-142 (26) x Clark 63 K	-0.12	-2.50	-0.48	-1.38	-0.67	-1.09
Pr-142 (26) x SCS-1	-0.36	-10.93	2.37	-3.13	-3.08	-25.30
Pr-142 (26) x G-9945	0.02	-0.62	-1.15	0.96	0.94	1.91
Pr-142 (26) x Crowford	-0.80	-19.84	1.02	-2.72	-2.18	-40.09
H-3 x Clark 63 K	-0.10	-2.90	0.38	1.60	0.39	-6.97
H-3 x SCS-1	0.58	3.35	2.70	1.85	2.31	24.19
H-3 x G-9945	-0.12	0.46	-1.46	-1.56	-0.72	0.16
H-3 x Crowford	0.11	-0.49	0.64	1.50	0.04	4.21
Clark 63 K x SCS-1	0.61	-3.90	-1.16	3.19	2.27	-4.14
Clark 63 K x G-9945	-1.34	-14.82	0.58	-5.47**	-4.78**	-45.72
Clark 63 K x Crowford	0.93	-2.24	1.59	1.59	3.30	-1.72
SCS-1 x G-9945	-0.21	-0.92	-0.04	0.78	0.94	1.69
SCS-1 x Crowford	-0.81	-4.34	-1.76	-1.90	-2.61	-25.29
G-9945 x Crowford	-1.14	-21.93	-1.90	-3.56	-3.56	-51.25
S.E	0.74	13.65	1.51	2.27	1.95	33.84

**Fig 2:**
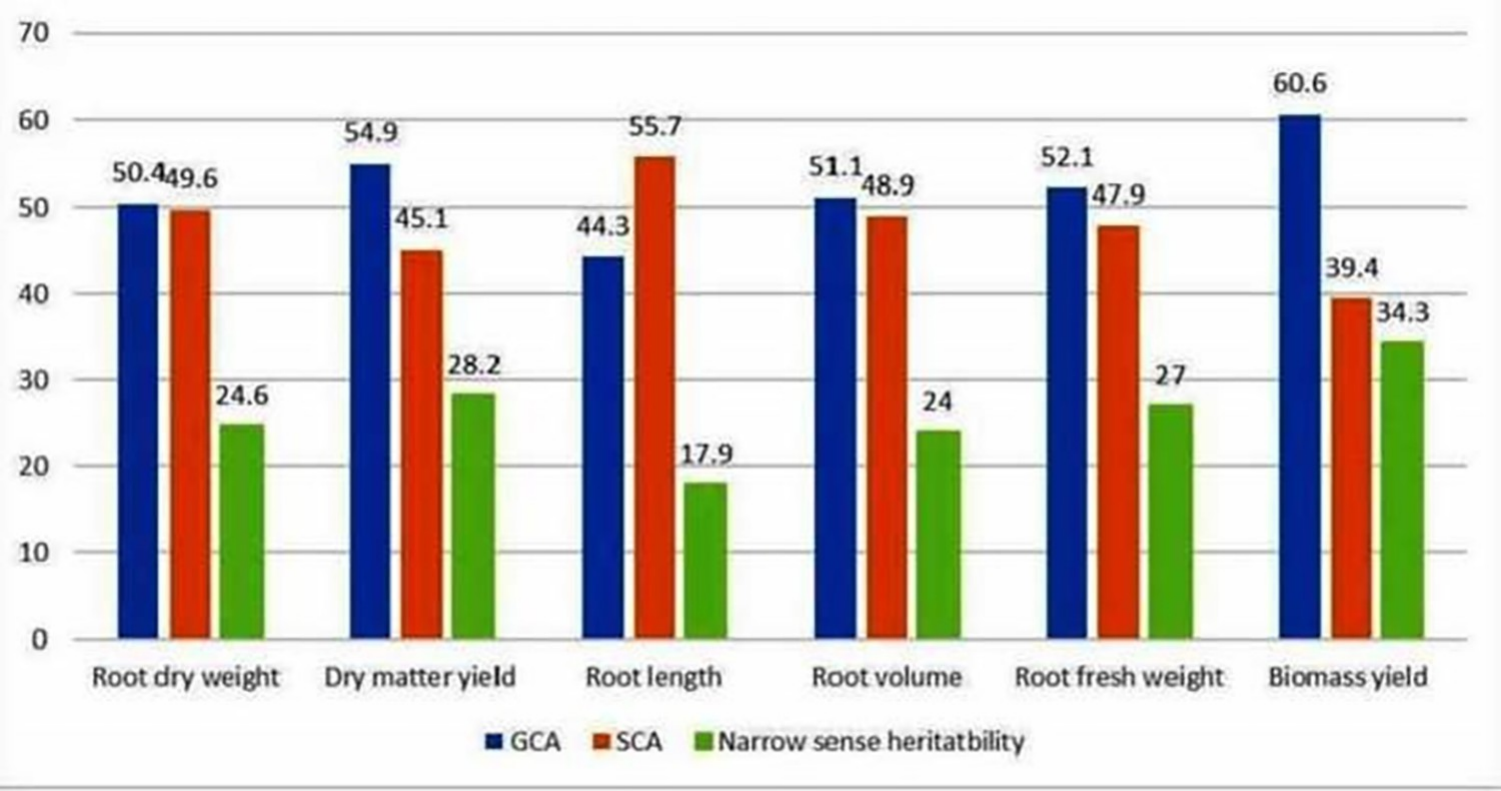
Relative contributions of GCA and SCA, and narrow-sense heritability of the studied biomass yield and root- related traits evaluated under low P conditions in western Ethiopia.

The relative contributions of GCA and SCA, and narrow sense heritabilities of the studied root and shoot biomass traits were presented in Fig 2. The study revealed the highest (60.6%) contribution of GCA over SCA to the inheritance of biomass yield, followed by biomass dry matter yield (54.9%). The relative contributions of SCA was higher over GCA variance for only root length (55.7%). Nearly equivalent contribution of GCA and SCA was found for root dry matter, root volume and root fresh weight. The highest narrow-sense heritability of 34.3% was recorded for biomass yield; while biomass dry matter yield, root fresh weight, root dry weight, root volume and root length showed heritabilities of 28.2%, 27.0%, 24.6%, 24.0%, and 17.9%, respectively.

**Fig 3:**
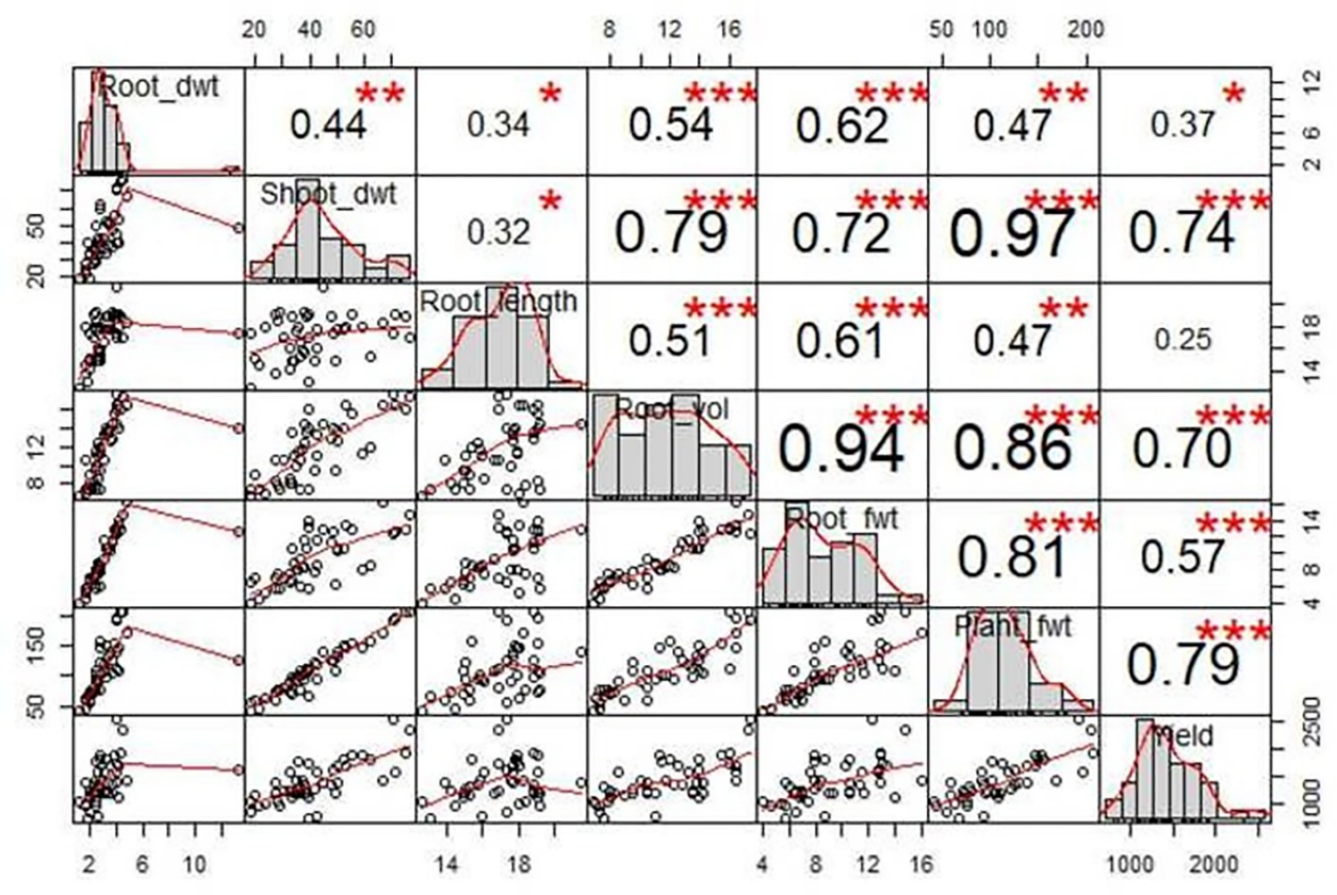
Correlation among root agronomic traits under low soil P conditions. NB: Root_dwt = root dry weight, shoot_dwt = shoot dry weight, root_vol = root volume, root_fwt = root fresh weight, plant_fwt = biomass yield.

The correlation analysis revealed a strong, highly significant positive association of grain yield with biomass yield (0.79), biomass dry matter yield (0.74), root weight (0.70), root fresh weight (0.57). Similarly, grain yield showed significant (P<0.05) association with root dry weight, while no association was found with root length (Fig 3). The correlations among all the rest of the traits were strong, and highly significant, except for the correlation of root length with root dry weight (0.34) and shoot dry weight (0.32) that were significant only at 5% level of significance.

## Discussion

The 6.07 and 8.40 ppm P levels recorded for Assosa and Mettu experimental sites, respectively, were far lower than the optimal P levels. The optimum level of P reported for soybeans was 15 ppm and soil P levels should be maintained between 15 and 30 ppm [35]. Accordingly, the two experimental sites (Assossa and Mettu) can be regarded as low P soils (soils that are deficient in P).

Significant differences observed among the parental genotypes as well as in the progeny populations for all the studied traits in the present study might be as a result of the high genetic variation created in the progeny populations that may allow the genetic improvement of the populations for the studied biomass yield and root related traits for low P tolerance. The presence of genetic variability is of prime importance for good genetic progress in improving a trait in a selection program. The use of parental genotypes from diverse sources in the crossing might have contributed to the observed significant differences and high genetic variations among the crosses and progeny populations. Similarly, highly significant differences among parents and progeny populations were also reported for grain yield, 100-seed weight, pod length, days to maturity and plant height under low P conditions [36]. Significant GCA and SCA effects were found for all the studied traits. Susanto [37] also reported similar significant GCA, and SCA effects for maturity in soybean, indicating both the additive and non-additive gene actions were important in the inheritance of the traits. The G X E interaction was significant (P<0.05) only for root length, implying the relative performance of the genotypes for root length was different in varying environments. The GCA X E interaction was higher than the Genotype X E interaction for all the studied traits, except for root length and root volume. Similar findings were previously reported for other traits in dry beans [38] and in soybean [36] under P stressed soil conditions that might indicate the predominant contributions of GCA than the environments in the expression of these traits. The highly significant GCA X E interaction for biomass dry matter yield and the significant GCA X E interaction for biomass yield indicates that more than one location was required for a reliable selection of parental lines to improve these biomass yield traits [39]. The non-significant GCA x E effects for root dry weight, root length, root volume and root fresh weight indicates that the GCA effects associated with the parents were consistent across environments. Thus, selection of these parents for progeny population and line development could be done in any of the environments. Similarly, the non-significant SCA x E interaction of the studied traits, except for root length suggested that the relative performance of the F2/F3 progeny populations was consistent across environments. The diallel analysis revealed highly significant GCA effects for biomass and biomass dry matter yield and root related traits. In line with this, highly significant GCA mean squares were reported for root dry weight, shoot dry weight and relative root surface area in soybean for Aluminium toxicity tolerance under acidic conditions [40]. Highly significant GCA effects were also previously reported for grain yield, pod length, days to maturity and plant height in soybean under low P conditions [36]. Similarly, the SCA effects were significant for all the root and biomass yield related traits. The GCA effects are associated with additive gene effect; while SCA effects were associated with non-additive gene action. Hence, the significance of both GCA and SCA effects for all the traits indicates both the additive and non-additive genetic effects were important for the expression of all the studied traits.

The relative importance of GCA and SCA was examined by expressing it as the ratio of additive variance to the total genetic variance. The closer this ratio was to unity, the greater the predictability based on GCA alone [33]. The ratio ranged from 44.3% for root length to 60.6% for biomass. The relative contributions revealed the predominance of GCA over SCA effects for biomass yield, biomass dry matter yield, root length, root fresh weight, indicating the predominance of additive genetic variance in the inheritance of these traits. This also implicates the high possible flow of favorable additive genes from parents to progenies, and also depicts high heritability, low environmental effect, less interaction among genes and, thereby, the effectiveness and predictability of selection to improve these traits [41–43]. The predominance of GCA over SCA also indicates early generation testing may be more effective in identifying promising progeny populations and breeding lines for biomass yield and root related traits, mainly based on the prediction from GCA effects under P stressed soil [43–45]. Considerable additive genetic variance was also reported for root traits in common bean under P stressed conditions [46]. The relative contributions of both GCA and SCA were nearly the same for root dry weight, root volume, and root fresh weight, which indicates both the additive and non-additive gene actions were equally important in the inheritance of these traits. In line with this [47], reported the complex nature of the inheritance of root traits and revealed comparable importance of both the additive and non-additive genetic variances in the expression of these traits in Capscicum under moisture stress conditions, and hence suggested recurrent selection is the effective breeding approach to improve these traits. The SCA effect showed high relative contribution over GCA for root length, which indicate the predominance of non-additive gene actions in the expression of this trait and, hence, selection will be relatively unpredictable. When non-additive gene actions are preponderant; selection needs to be performed at later generations, when the effects of the non-additive gene effects get fixed [43, 48, 49].

Crowford was identified as the best performing parent based on the mean values for most traits, followed by SCS-1 and Hardee-1. GCA effects for all the traits varied significantly among the parental genotypes and between environments for biomass dry matter yield and biomass yield. Hardee-1 showed significant positive GCA effects for biomass dry matter yield, root volume and biomass yield; while Alamo displayed significant negative GCA effects for root dry weight, root length, root volume, root fresh weight and biomass yield. In a recent study, Hardee-1 was reported possessing significant positive GCA effects for grain yield, number of seeds per pod, pod length, plant height and pod number; while Alamo showed significant negative GCA effects for hundred seed weight, pod length, and plant height [36]. Hardee-1 was identified as the best general combiner for root traits, grain and forage yield, and root related traits was capable of contributing favorable alleles and, hence, could be used as parents in a breeding program to improve the respective traits under low P conditions. Susanto [37] also reported Grobogan and Malabar soybean varieties as the best general combiner parents for early maturity. Of the top ten performing F2/F3 progenies, seven were derived from crosses involving the parent Hardee-1 that was the best general combiner with significant positive GCA effects with other poor general combiners for most of the traits. The high SCA effect of the good by poor general combiner crosses were attributed either to the favourable additive gene effects of the good general combiner parent or to the epistatic effects of poor general combiner that fulfils the favourable plant attribute [43]. Conversely, five out of the ten worst performing F2/F3 progenies were derived from crosses involving Alamo that displayed significant negative GCA effects for most of the studied traits.

Average heritability estimates were categorized as high, medium and low with respective values of 5–10%, 10–30%, and 30 to 60%, that represent different crops and across locations and seasons [50, 51]. Accordingly, biomass yield was the only trait that possessed high narrow sense heritability, while the narrow sense heritabilities of all the rest of the traits i.e., biomass dry matter yield, root fresh weight, root dry weight, root volume, and root dry weight might be regarded as medium. The highly significant and positive association of grain yield with biomass yield, biomass dry matter yield, root volume, and root fresh weight indicates the importance of these traits for indirect selection to improve yield of soybean under P stressed soil conditions. Moreover, the strong positive association of biomass and grain yield in soybean imply the possibility of developing dual purpose soybean that can serve both as a forage or grain soybean. In line with this [3], reported that the standard grain type soybean varieties produced optimum forage yield and quality than the soybean varieties exclusively developed for forage purpose. This indicates that the regular grain soybean breeding programs can simultaneously target developing dual purpose soybean for grain as well as forage purposes. Hence, considering biomass yield as one of the priority traits for breeding soybean will have paramount significance in developing dual purpose soybean.

## Conclusions

A half-diallel experiment involving nine parental genotypes and their F2/F3 progeny populations was conducted at two environments under low P soil conditions. The results revealed wide genetic variability among the parental genotypes and progeny populations used in the study. Both the GCA and SCA effects were significant, indicating the importance of both the additive and non-additive gene actions in the inheritance of the traits. Among the parents, Hardee-1 was the best general combiner for root traits, yield and yield-related traits, and hence the best parent to improve soybean for low P tolerance. High narrow sense heritability and high relative contributions of GCA over SCA was found for biomass yield, indicating the importance of additive gene effects in the inheritance of this trait, and recurrent selection in the segregating population would be the best approach to improve the trait for low-P tolerance. The high and positive correlation of biomass yield with grain yield indicates that improving biomass yield can help improve the productivity of soybean under low P conditions. More importantly, soybean biomass can greatly help improve the forage yield and value of soybean as an alternative animal feed to Alfaalfa and other forage crops.

## References

[1] Teodoro LPR, Bhering LL, Teodoro PE. Understanding the combining ability for physiological traits in soybean. PLoS One 2019, 14 (12): e0226523. doi: 10.1371/journal.pone.0226523 31846491 PMC6917344

[2] Goplen J, Sheaffe C, Drewitz N, Mousel E, Martinson K, Salfer J, et al. Soybeans as forage. Minnesota crop news, University of Minnesota, Extension, 2021. Available from: https://blog-crop-news.extension.umn.edu/2021/08/soybeans-as-forage.html.

[3] Wiederholt R, Albrech K. Using Soybean as Forage. University of Wisconsin Board of Regent, University of Wisconsin Extension. Focus on Forage 2003, 5 (13) 1–2. Available from: https://fyi.extension.wisc.edu/forage/files/2014/01/SoybeanForageFOF.pdf.

[4] Blount AR, Wright DL, Sprenkel RK, Hewitt TD, Myer RO. Forage soybeans for grazing, hay, and silage. University of Florida, IFAS Extension. SS-AGR-180; 1–4, 2017. Available from: https://edis.ifas.ufl.edu/publication/AG184.

[5] Salvagiotti F, Cassman KG, Specht JE, Walters DT, Weiss A, Dobermann AR. Nitrogen uptake, fixation and response to fertilizer N in soybeans: A review. Field Crops Res. 2008, 108 (1) 1–13. 10.1016/j.fcr.2008.03.001.

[6] Jensen ES, Peoples MB, Hauggaard-Nielsen H. Faba bean in cropping systems. Field Crops Res. 2010, 115 (3): 203–216. 10.1016/j.fcr.2009.10.008.

[7] Köpke U, Nemecek T. Ecological services of faba bean. Field Crops Res. 2010, 115 (3): 217–233. doi: 10.1016/j.fcr.2009.10.012

[8] Xia H, Zhao J, Sun J, Xue Y, Eagling T, Bao X, et al. Maize grain concentrations and above-ground shoot acquisition of micronutrients as affected by intercropping with turnip, faba bean, chickpea, and soybean. Sci. China Life Sci. 2013, 56 (9) 823–834. doi: 10.1007/s11427-013-4524-y 23900569

[9] Cong WF, Hoffland E, Li L, Six J, Bao XG, Zhang FS, et al. Intercropping enhances soil carbon and nitrogen. Global Change Biol. 2015, 21, 1715–1726. doi: 10.1111/gcb.12738 25216023

[10] Zhou T, Du Y, Ahmed S, Liu T, Ren M, Liu W, et al. Genotypic differences in phosphorus efficiency and the performance of physiological characteristics in response to low phosphorus stress of soybean in Southwest of China. Front. Plant Sci. 2016, 7: 1776. doi: 10.3389/fpls.2016.01776 27933086 PMC5121124

[11] Liao H, Wan H, Shaff J, Wang X, Yan X, Kochian LV. Phosphorus and Aluminum Interactions in Soybean in Relation to Aluminum Tolerance. Exudation of Specific Organic Acids from Different Regions of the Intact Root System. Plant Physiol. 2006, 141 (2) 674–684. doi: 10.1104/pp.105.076497 16648222 PMC1475464

[12] Abebe M. Nature and management of acid soils in Ethiopia. Retrieved from: -Acid- Management, 2007. Available from: http://www.scribd.com/doc/134036957/Soils.

[13] Gurmessa B. Soil acidity challenges and the significance of liming and organic amendments in tropical agricultural lands with reference to Ethiopia. Environ. Dev. Sustain. 2020, 1–23. 10.1007/s10668-020-00615-2.

[14] Serra AP, Marchetti ME, Dupas E, Carducci CE, da Silva EF, Pinheiro ER. Phosphorus in Forage Production. In: Ricardo Loiola Edvan and Leilson Rocha Bezerra (Eds.). New Perspectives in Forage Crops, 2017. doi: 10.5772/intechopen.70202

[15] Silva G. The peaks and valleys of phosphorus fixation. Michigan State University Extension. March 30, 2012. Available from: https://www.canr.msu.edu/news/the_peaks_and_valleys_of_phosphorus_fixation#:~:text=Phosphorus%20(P)%20fixation%20happens%20when,to%20start%20is%20soil%20pH. Accessed date: 19/04/2022.

[16] Zhang D, Song H, Cheng H, Hao D, Wang H, Kan G, et al. The Acid Phosphatase- Encoding Gene GmACP1 Contributes to Soybean Tolerance to Low-Phosphorus Stress. PLoS Genet 2014, 10 (1): e1004061. doi: 10.1371/journal.pgen.1004061 24391523 PMC3879153

[17] Usherwood N.R. Nutrient management for top-profit soybeans, 1998. www.ppi-ppic.org.

[18] Suman B. Nutrient Management for Soybean Crops. International Journal of Agronomy, Hindawi 2021, Article ID 3304634, 10 pages. URL: 10.1155/2021/3304634.

[19] Khanam M, Islam MS, Ali MH, Chowdhury IF, Masum SM. Performance of soybean under different levels of phosphorus and potassium. Bangladesh Agron. J. 2016, 19 (1): 99–108. doi: 10.3329/baj.v19i1.29876

[20] DAFF. Soybeans production guideline. Department of Agriculture, Forestry and Fisheries, RSA, DPP, 2010. Available from: daff.gov.za.

[21] Sheaffer CC, Orf JH, Devine TE, Jewett JG. Yield and Quality of Forage Soybean. Agron. J. 2001, 93:99–106. doi: 10.2134/agronj2001.93199x

[22] Lynch JP. Root phenotypes for improved nutrient capture: an underexploited opportunity for global agriculture. New Phytol. 2019, 223, 548–564. doi: 10.1111/nph.15738 30746704

[23] Schneider HM, Lynch JP. Should Root Plasticity be a Crop Breeding Target? Front. Plant Sci. 2020, 11:546. doi: 10.3389/fpls.2020.00546 32499798 PMC7243933

[24] Rangarajan H, Postma JA, Lynch J. Co-optimisation of axial root phenotypes for nitrogen and phosphorus acquisition in common bean. Ann. Bot. 2018, 122, 485–499. doi: 10.1093/aob/mcy092 29982363 PMC6110351

[25] Woods J, Williams A, Hughes JK, Black M, Murphy R. Energy and the food system. Philos. Trans. R. Soc. B: Biol. Sci. 2010, 365: 2991–3006. doi: 10.1098/rstb.2010.0172 20713398 PMC2935130

[26] Fried HG, Narayanan S, Fallen B. Characterization of a soybean (*Glycine max* L. Merr.) germplasm collection for root traits. PLoS One; 2018, 13 (7): e0200463. doi: 10.1371/journal.pone.0200463 29995945 PMC6040769

[27] Gomez KA, Gomez A.A. Statistical Procedures for Agricultural Research. 2nd Edition, John Wiley and Sons, New York, 1984, 680 p.

[28] Statistical Analysis Systems, SAS. The SAS System for Windows, Release 9.4. Statistical Analysis Systems Institute, Cary, NC, 2013.

[29] Zhang Y, Kang MS, Kendall RL. DIALLEL-SAS05: a comprehensive programme for Griffing’s and Gardner–Eberhart Analyses. Agron. j. 2005, 97:1097–1106. 10.2134/agronj2004.0260.

[30] Farshadfar E, Rafiee F, Yghotipoor A. Comparison of the efficiency among half diallel methods in the genetic analysis of bread wheat (*Triticum aestivum* L.) under drought stress condition. Scholars Res. Lib. Ann. Biol. Res 2012, 3 (3): 1607–1622. Available from: http://scholarsresearchlibrary.com/archive.html.

[31] Singh KB, Chaudhary BD. Biometrical methods in quantitative genetic analysis. Kalyani Publishers, New Delhi, India, 1977, pp304.

[32] Hasanuzzaman M, Hakim MA, Fersdous J, Islam MM, Rahman L. Combining ability and heritability analysis for yield and yield contributing characters in Chilli (Capsicum annuum) landraces. Plant Omics J. 2012, 5(4):337–344.

[33] Baker RJ. Issues in diallel analysis. Crop Sci. 1978, 18: 533–536.

[34] Hung HY, Holland JB, Diallel analysis of resistance to Fusarium ear rot and fumonisin contamination in maize. Crop Sci. 2012, 52: 2173–2181. doi: 10.2135/cropsci2012.03.0154

[35] Staton M. Phosphorus and potassium fertilizer recommendations for high-yielding, profitable Soybeans. Michigan State University Extension, 2014. Available from: http://msue.anr.msu.edu/news/phosphorus_and_potassium_fertilizer_recommendations_f or_high_yielding_profi, Accessed October 2, 2021.

[36] Abebe AT, Githiri M, Derera J, Debele T. Combining ability of soybean (*Glycine max*) for low phosphorus tolerance on acidic soils of Western Ethiopia. Plant Breed. 2020, 139 (4). doi: 10.1111/pbr.12817

[37] Susanto GWA. Estimation of gene action through combining ability for maturity in soybean. SABRAO J. Breed. Genet., 2018, 50 (1) 62–71.

[38] Kimani JM, Derera J. Combining ability analysis across environments for some traits in dry bean (*Phaseolus vulgaris* L.) under low and high soil phosphorus conditions. Euphytica 2009, 166 (1) 1–13.

[39] Patil VD, Chopde PR. Combining ability analysis over environments in diallel crosses of linseed (*Linum usitatissimum* L.). Theor. Appl. Genet. 1981, 60: 339–343. doi: 10.1007/BF00264325 24276920

[40] Ojo GOS, Ayuba SA. Combining ability and heterosis for aluminum stress tolerance of soybean roots and shoots grown in acid sand. J. Plant Breed. Crop Sci. 2013, 5 (1) 6–11. doi: 10.5897/JPBCS12.038

[41] Topal A, Aydın C, Akgun N, Babaoglu M. Diallel cross analysis in durum wheat (*Triticum durum* Desf.): identification of best parents for some kernel physical features. Field Crops Res 2004, 87: 1–12.

[42] Chigeza G, Mashingaidze K, Shanahan P. Advanced cycle pedigree breeding in sunflower. II: combining ability for oil yield and its components. Euphytica 2014, 195 (2): 183–195.

[43] Fasahat P, Rajabi A, Rad JM, Derera J. Principles and utilization of combining ability in plant breeding. Biom. biostat. int. j. 2016, 4 (1):1‒22. doi: 10.15406/bbij.2016.04.0008

[44] Melchinger A, Schmit W, Geiger HH. Comparison of testcrosses from F2 and first backcross populations in maize. Crop Sci. 1998, 28 (5): 743–749.

[45] Smith JSC, Hussain T, Jones ES, Graham G, Podlich D, Wall S, et al. Use of doubled haploids in maize breeding: implications for intellectual property protection and genetic diversity in hybrid crops. Mol. Plant Breed 2008, 22 (1): 51–59.

[46] Araujo AP, Antunes IF, Teixeira MG. Inheritance of root traits and phosphorus uptake in common bean (*Phaseolus vulgaris* L.) under limited soil phosphorus supply. Euphytica 2005, 145: 33–40. doi: 10.1007/s10681-005-8772-1

[47] Naresh P, Bhatt RM, Venkatachalapathi V, Gangadhararao P, Reddy KM. Inheritance of Root Traits in an Interspecific Cross of *Capsicumw annuum* × C. *chinens* in the Presence of Low Moisture. Int. J. Veg. Sci. 2017, 23 (6) 575–583. doi: 10.1080/19315260.2016.1221016

[48] Yin-guang B, Sen W, Xiu-qin W, Yu-hai W, Xing-feng L, Lin W, et al. Heterosis and combining ability for major yield traits of a new wheat germplasm Shannong 0095 derived from Thinopyrum intermedium. Agri. Sci. in China. 2009, 8 (6): 753–760.

[49] Zeng L, Pettigrew WT. Combining ability, heritability, and genotypic correlations for lint yield and fiber quality of upland cotton in delayed planting. Field Crops Res. 2015, 171: 176–183.

[50] Robinson HF. Quantitative genetics in relation to breeding of the centennial of mendalism. Indian J Genet 1966, 26: 171–187.

[51] Dabholkar AR. Elements of biometrical genetics. Concept Publishing Company, New Delhi, India. 1992, pp 431.

